# Supplementary material for: Pseudomonas aeruginosa Dnr-regulated denitrification in microoxic conditions
Source: Microbiol Spectr. 2025 Aug 7;13(9):e00682-25. doi: 10.1128/spectrum.00682-25 (PMC12403760; doi:10.1128/spectrum.00682-25)
Supplement: Supplemental figures and tables — Fig. S1 to S7 and Tables S1 and S2. [file spectrum.00682-25-s0001.pdf]

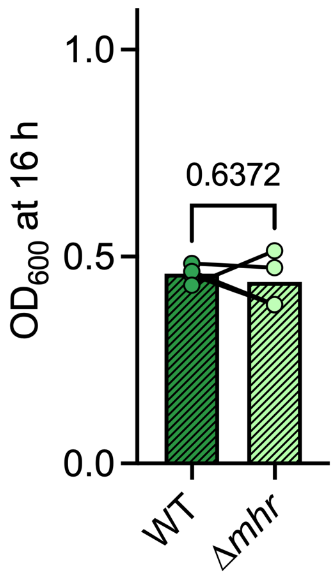

Figure S1. Anr-regulated *mhr* contribution to microoxic growth in LB. WT and Δ*mhr* growth at 1% O_2 in LB in a 96-well plate for 16 h with shaking. Each data point represents an average of replicates from one day with lines connecting data from the same day. P-values were calculated using a paired t-test.

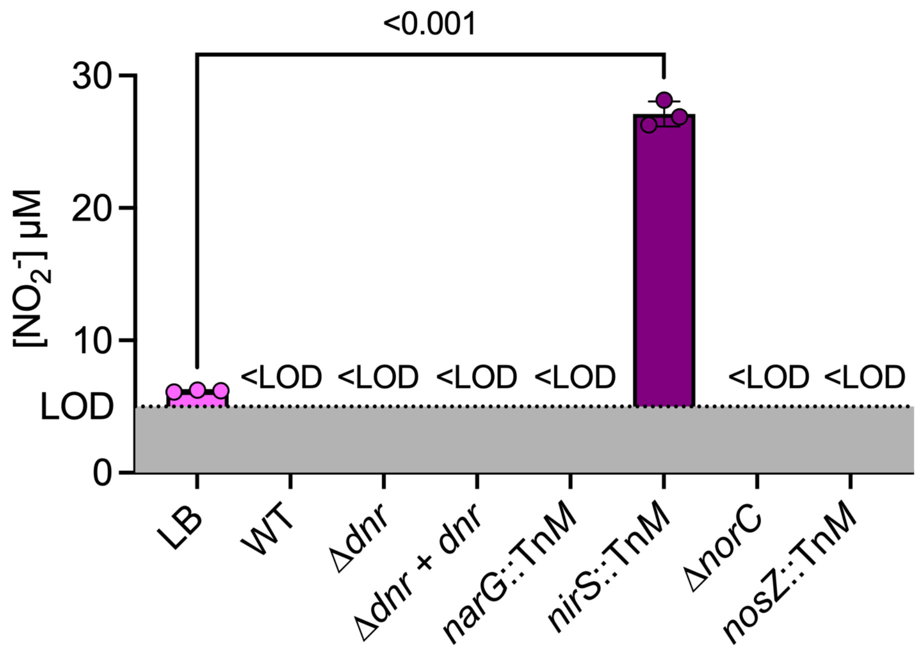

Figure S2. Levels of nitrite in LB and supernatants. Concentration of nitrite (NO_2) in LB and in supernatants after 16 h of growth of indicated strains in 5 mL culture tubes. Concentrations were calculated using a standard curve of sodium nitrite $NaNO_2$ in water. Levels below the limit of detection (LOD) are considered not detected (n.d.). Each data point represents a biological replicate, and the P-value was calculated using an unpaired t-test.

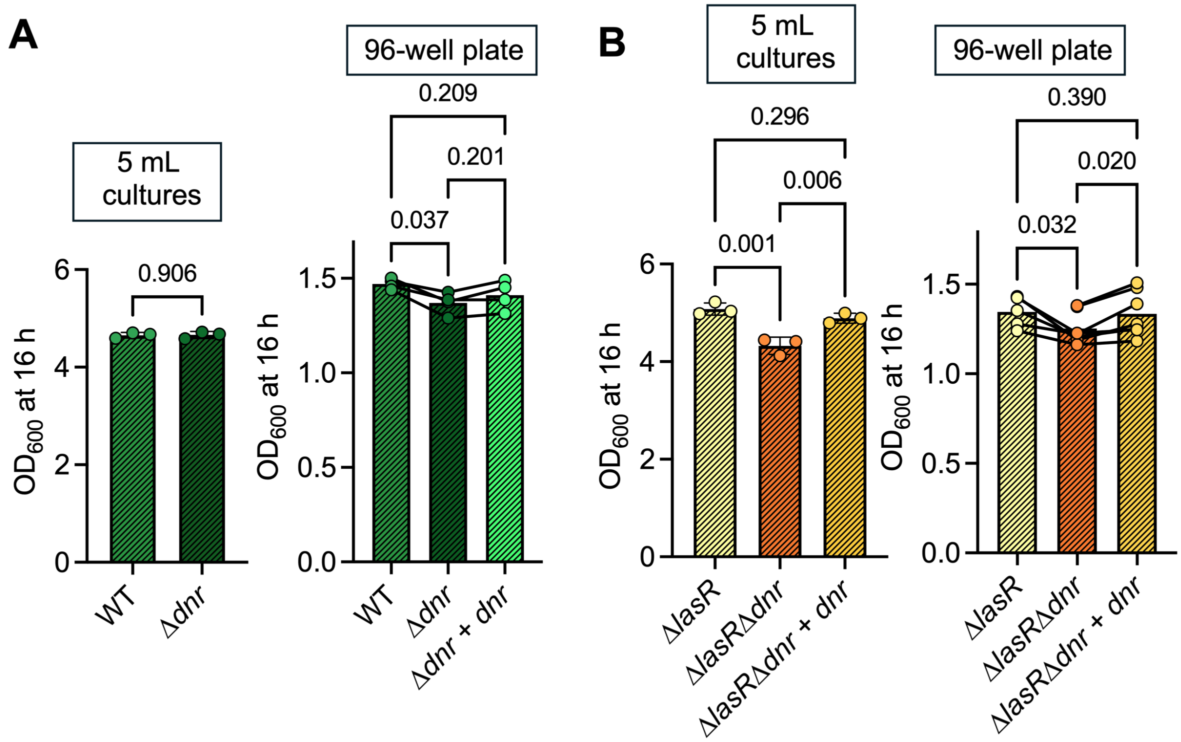

Figure S3. Normoxic growth of *P. aeruginosa*. OD_{600} of WT, Δ*dnr*, and Δ*dnr*+*dnr* (**A**) and Δ*lasR,* Δ*lasR*Δ*dnr* and Δ*lasR*Δ*dnr*+*dnr* (**B**) cultures grown in 5 mL LB on a roller drum, or 200 µL LB in a 96-well plate for 16 h on a shake plate at 21% O_2. Data points from 5 mL cultures each represent a biological replicate, data points from 96-well plates represent an average of replicates from one day with lines connecting data from the same day. P-values were calculated using an unpaired t-test (A, 5 mL culutres), an ordinary one-way ANOVA with multiple comparisons (B, 5 mL cultures), or a paired one-way ANOVA with multiple comparisons (96-well plates).

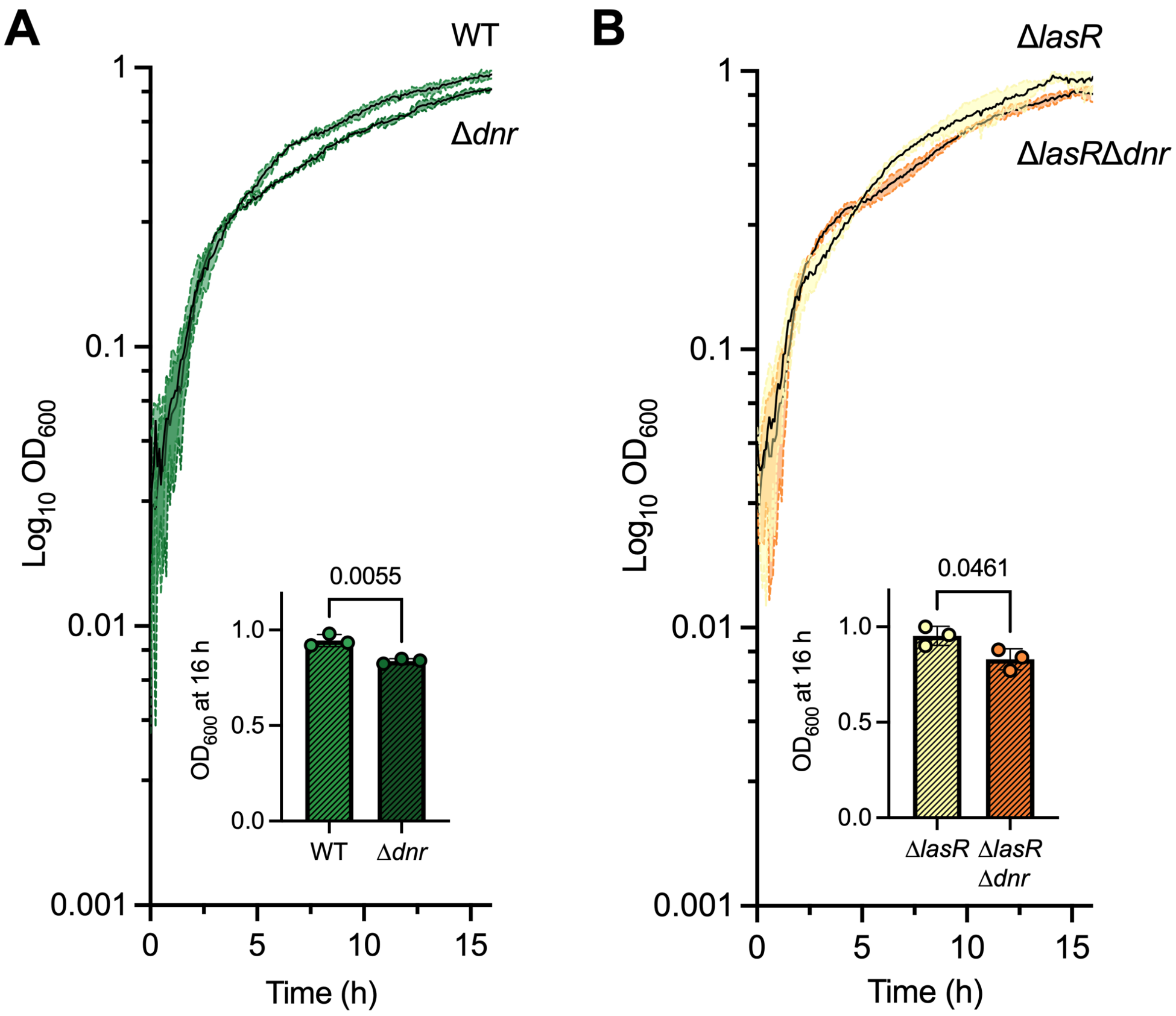

Figure S4. Growth curves of WT and Δ*lasR* in microoxia. The growth curves for the WT strain and Δ*dnr* mutant (**A**) as well as the Δ*lasR* and Δ*lasR*Δ*dnr* mutants (**B**) in LB in a 96-well plate for 16 h at 1% O_2 are shown. Each data point is an average of three replicates from one day. Similar results were obtained on three different days. P-values were calculated using a paired t-test.

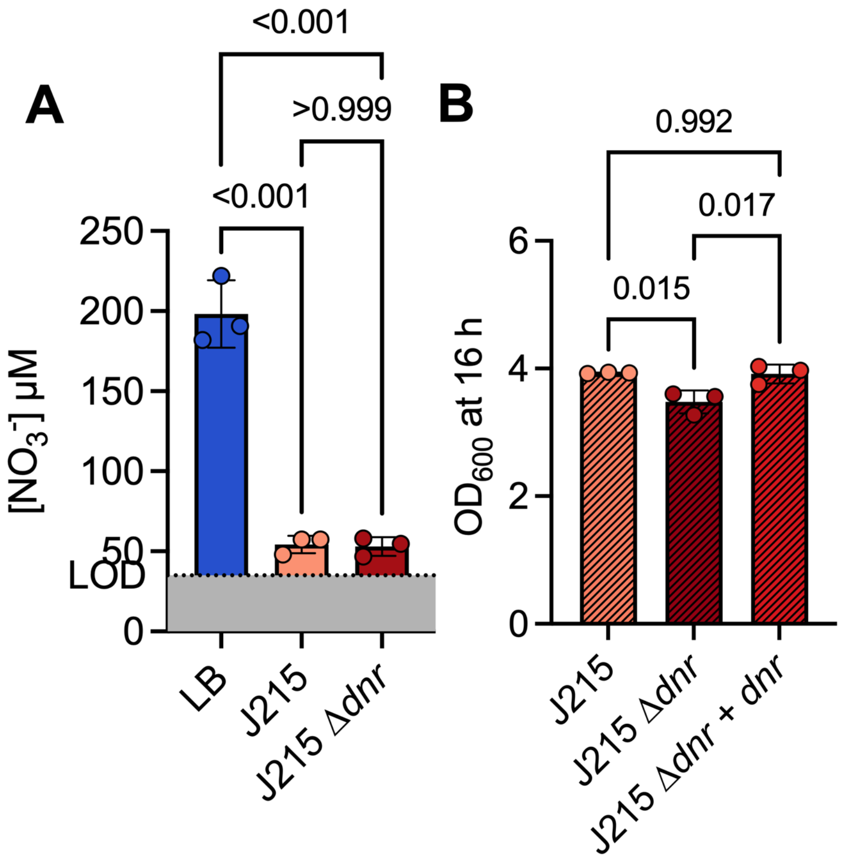

Figure S5. Nitrate consumption and growth of *P. aeruginosa* strain J215. A. The levels of nitrate in LB before and after 16 h of growth of the J215 strain and J215 Δ*dnr* mutant at 21% O_2 for 16 h. NO_3^- levels were calculated using a standard curve of KNO_3 in water and normalized to OD_{600}. Nitrate levels in LB are the same as in Figure 1B. **D.** Growth after 16 h of of J215, Δ*dnr* mutant and Δ*dnr*+*dnr* strain in 5 mL LB cultures. Data points each represent a biological replicate. P-values were calculated using an ordinary one-way ANOVA with multiple comparisons.

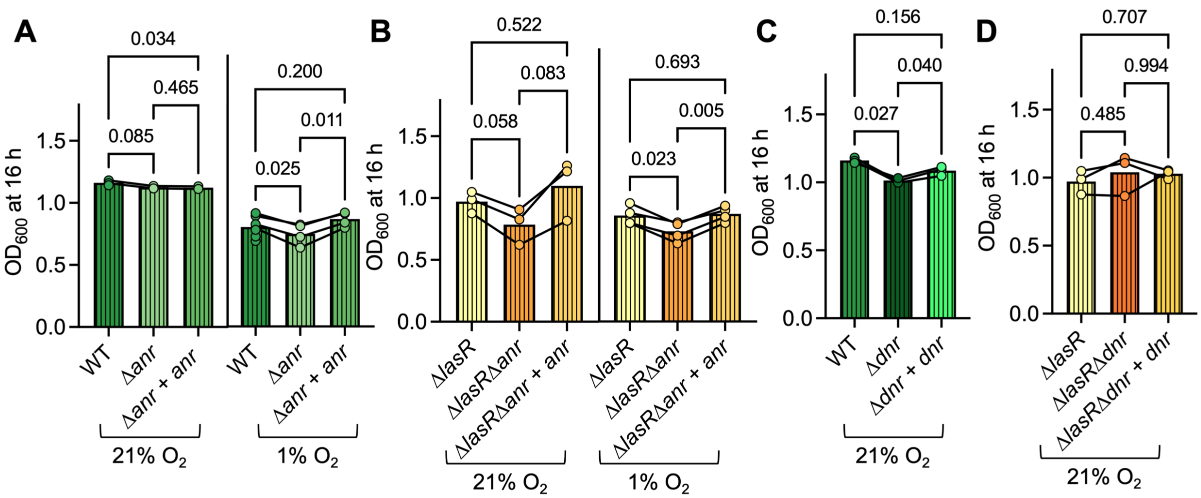

Figure S6. Anr- and Dnr-dependent growth of *P. aeruginosa* in ASMi. A. Culture densities for WT, Δ*anr* mutant, and the Δ*anr*+*anr* strain in ASMi at 21% and 1% O_2 in a 96-well plate after 16 h. **B.** The culture densities of Δ*lasR*, Δ*lasR*Δ*anr*, and the Δ*lasR*Δ*anr*+*anr* strains grown in ASMi at 21% and 1% O_2 in a 96-well plate for 16 h. **C.** Growth of WT, Δ*dnr* mutant, and Δ*dnr*+*dnr* strains in ASMi after 16 h in a 96-well plate at 21% O_2. **D.** Culture densities of the Δ*lasR*, Δ*lasR*Δ*dnr* and the Δ*lasR*Δ*dnr*+*dnr* strain in ASMi in 96-well plates at 21% O_2. All 96-well plates were grown with shaking. Each data point represents an average of replicates from one day with lines connecting data from the same day. All P-values were calculated using a paired one-way ANOVA with multiple comparisons.

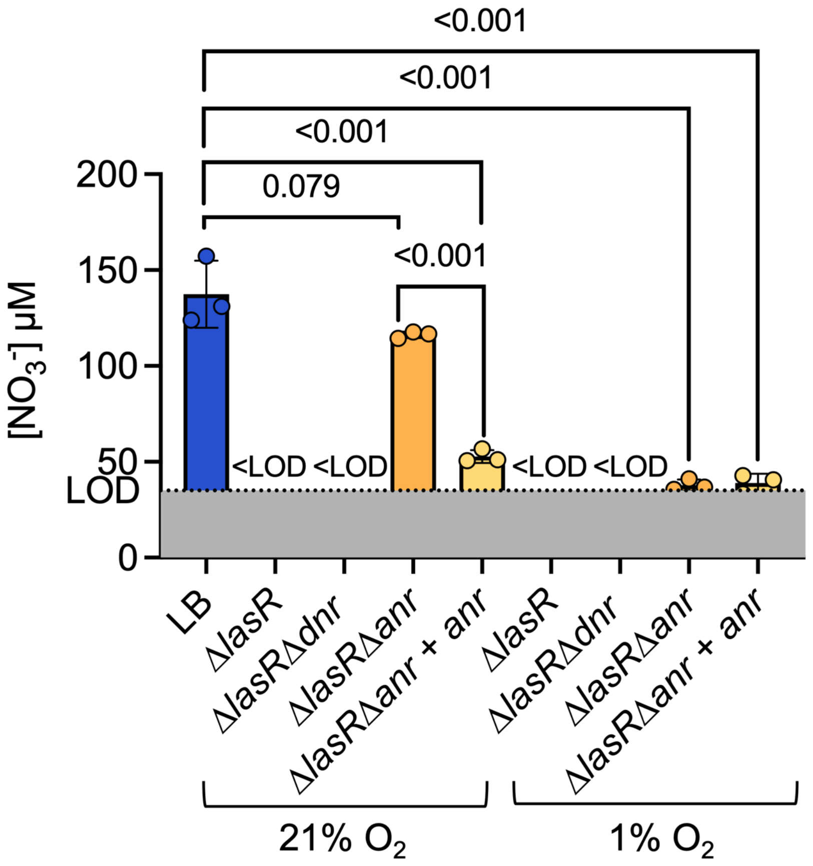

Figure S7. Nitrate consumption and growth of *P. aeruginosa* Δ*lasR* mutant. The levels of nitrate in LB before and after 16 h of growth of the Δ*lasR*, Δ*lasR*Δ*dnr*, Δ*lasR*Δ*anr* mutants and a Δ*lasR*Δ*anr*+*anr* strain at 21% and 1% O_2 for 16 h. NO_3^- levels were calculated using a standard curve of KNO_3 in water and normalized to OD_{600}. Levels of nitrate in LB are the same as in Figure 1B, levels of nitrate in Δ*lasR* supernatant at 21% and 1% O_2 are the same as in Figure 2A. Each data point represents a biological replicate. P-values were calculated using an ordinary one-way ANOVA.

Table S1. Strains and plasmids used in this study

Strain or plasmid	Strain ID	Description	Source
P. aeruginosa			
PA14 WT	DH122	Wild-type *P. aeruginosa*	(1)
Δ*anr*	DH2855	DH122 with in frame deletion of *anr* (PA14_44490)	(2)
Δ*anr*+*anr*	DH3478	DH2855 with complementation of *anr* (PA14_44490) at the native locus	(2)
Δ*dnr*	DH1978	In-frame deletion of *dnr*	
Δ*dnr att*Tn7::*dnr*	DH4598	DH1978 with complementation of *dnr* at the Tn*7* attachment (*att*Tn7) site, GmR	This study
Δ*lasR*	DH164	DH122 with in frame deletion of *lasR* (PA14_45960)	(3)
Δ*lasR*Δ*anr*	DH2401	In frame deletions of *lasR* (PA14_45960) and *anr* (PA14_44490)	(4)
Δ*lasR*Δ*anr*+*anr*	DH3479	DH2401 with complementation of *anr* at the native locus	(5)
Δ*lasR*Δ*dnr*	DH3813	In-frame deletion of *lasR* (PA14_45960) in DH1978	This study
Δ*lasR*Δ*dnr* *att*Tn7::*dnr*	DH4599	DH3813 with complementation of *dnr* at the *att*Tn7site, GmR	This study
J215	DH2403	Clinical isolate J215 (with a non-functional LasR)	(4)
J215Δ*dnr*	DH2409	DH2403 with in frame deletion of *dnr* (PA14_45960)	This study
J215Δ*dnr* *att*Tn7::*dnr*	DH4602	DH2409 with complementation of *dnr* at the *att*::Tn*7* site, GmR	This study
narG::Tn*M*	DH1754	Transposon insertion in *narG* (gene), GmR	(6)
narK1::Tn*M*	DH1755	Transposon insertion in *narK1* (gene), GmR	(6)
narK2::Tn*M*	DH1756	Transposon insertion in *narK2* (gene), GmR	(6)
narX::Tn*M*	DH1757	Transposon insertion in *narX* (gene), GmR	(6)
nirS::Tn*M*	DH1758	Transposon insertion in *nirS* (gene), GmR	(6)
norB::Tn*M*	DH4601	Transposon insertion in *norB* (gene), GmR	(6)
Δ*norC*	DH4664	DH122 with in frame deletion of *norC* (PA14_06810)	This study
nosZ::Tn*M*	DH1760	Transposon insertion in *nosZ* (gene), GmR	(6)
Δ*norC* *att*Tn7::*norC*	DH4750	DH4664 with in frame complementation of *norC* at the *att*::Tn7 site, GmR	This study
E. coli			
SM10 + mini Tn*7*-*dnr*	DH4600	*E. coli* strain SM10 containing the plasmid to complement *dnr* for conjugation into *P. aeruginosa*, GmR	(7)

S17 + mini Tn7-*norC*	DH4749	*E. coli* strain S17 containing the plasmid to complement *norC* for conjugation into *P. aeruginosa*, GmR	This study
S17 + pMQ30_Δ*norC*	DH4743	*E. coli* strain S17 containing the plasmid for in-frame deletion of *norC* (PA14_06810)	This study
**Plasmids**			
puc18T-mini-Tn*7*T-Gm		*att*Tn*7* site insertion vector, GmR	(8)
puc18T-mini-Tn*7-dnr*		plasmid to complement *dnr* at *att*Tn*7* site in *P. aeruginosa*, GmR	(7)
puc18-mini-Tn7-*norC*		Plasmid to complement *norC* at *att*Tn*7* site in *P. aeruginosa*, GmR	This study
pMQ30		Allelic replacement vector, GmR	(9)
pMQ30_Δ*norC*		Plasmid for in-frame deletion of *norC*, GmR	This study

Table S2. Primers used in this study

Primer	Primer No.	Description
Arb1	DH179	Arbitrary primer 1 (5'-GGCCACGCGTCGACTAG TACGGNNNNNNNNNNGATAT-3')
Arb2	DH180	Arbitrary primer 2 (5'-GGCCACGCGTCGACTAG TACNNNNNNNNNNACGCC-3')
Arb6	DH181	Arbitrary primer 6 (5'-GGCCACGCGTCGAC TAGTACGGNNNNNNNNNNACGCC-3')
PMFLGM.GB-3a	DH115	Tn*M*-specific primer Round 1 (5'-TACAGTTTA CGAACCGAACAGGC-3')
PMFLGM.GB-2a	DH114	Tn*M*-specific primer Round 2 (5'-TGTCAACTG GGTTCGTGCCTTCATCC-3')
PMFLGM.GB-4a	DH116	Tn*M*-specific sequencing primer (5'-GACCGAGATAGGGTTGAGTG-3')
norC_down_HindIII	SB1	Amplification of *norC* for cloning into puc18T-mini-Tn7 (5'-GAGACAAGCTTCAACCCTCCTTGTTCGGCG GC-3')
norC_up_BamHI	SB2	Amplification of *norC* for cloning into puc18T-mini-Tn7 (5'-GAGACGGATCCGTGGCCAAGGGCTGGCTGA TC-3')

References

1. Rahme LG, Stevens EJ, Wolfort SF, Shao J, Tompkins RG, Ausubel FM. 1995. Common virulence factors for bacterial pathogenicity in plants and animals. Science 268:1899-902.
2. Crocker AW, Harty CE, Hammond JH, Willger SD, Salazar P, Botelho NJ, Jacobs NJ, Hogan DA. 2019. *Pseudomonas aeruginosa* ethanol oxidation by AdhA in low-oxygen environments. J Bacteriol 201. 23:e00393-19.
3. Hogan DA, Vik A, Kolter R. 2004. A *Pseudomonas aeruginosa* quorum-sensing molecule influences *Candida albicans* morphology. Mol Microbiol 54:1212-23.
4. Hammond JH, Dolben EF, Smith TJ, Bhuju S, Hogan DA. 2015. Links between Anr and Quorum Sensing in *Pseudomonas aeruginosa* Biofilms. J Bacteriol 197:2810-20.
5. Clay ME, Hammond JH, Zhong F, Chen X, Kowalski CH, Lee AJ, Porter MS, Hampton TH, Greene CS, Pletneva EV, Hogan DA. 2020. *Pseudomonas aeruginosa lasR* mutant fitness in microoxia is supported by an Anr-regulated oxygen-binding hemerythrin. Proc Natl Acad Sci U S A 117:3167-3173.
6. Liberati NT, Urbach JM, Miyata S, Lee DG, Drenkard E, Wu G, Villanueva J, Wei T, Ausubel FM. 2006. An ordered, nonredundant library of *Pseudomonas aeruginosa* strain PA14 transposon insertion mutants. Proc Natl Acad Sci U S A 103:2833-8.

7. Jackson AA, Daniels EF, Hammond JH, Willger SD, Hogan DA. 2014. Global regulator Anr represses PlcH phospholipase activity in *Pseudomonas aeruginosa* when oxygen is limiting. Microbiology (Reading) 160:2215-2225.
8. Choi K-H, Schweizer HP. 2006. mini-Tn7 insertion in bacteria with single *attTn7* sites: example *Pseudomonas aeruginosa*. Nat Protoc 1:153-161.
9. Shanks RM, Caiazza NC, Hinsa SM, Toutain CM, O'Toole GA. 2006. *Saccharomyces cerevisiae*-based molecular tool kit for manipulation of genes from gram-negative bacteria. Appl Environ Microbiol 72:5027-36.
